# CRB3 downregulation confers breast cancer stem cell traits through TAZ/β-catenin

**DOI:** 10.1038/oncsis.2017.24

**Published:** 2017-04-24

**Authors:** P Li, Y Wang, X Mao, Y Jiang, J Liu, J Li, J Wang, R Wang, J She, J Zhang, J Yang, Y Liu, P Liu

**Affiliations:** 1 Center for Translational Medicine, The First Affiliated Hospital of Xi’an Jiaotong University, Xi’an, Shaanxi, China; 2 Key Laboratory for Tumor Precision Medicine of Shaanxi Province, The First Affiliated Hospital of Xi’an Jiaotong University, Xi’an, Shaanxi, China; 3 Department of Pathology, The First Affiliated Hospital of Xi’an Jiaotong University, Xi’an, Shaanxi, China; 4 Department of Vascular Surgery, The First Affiliated Hospital of Xi’an Jiaotong University, Xi’an, Shaanxi, China; 5 Department of General Surgery, The First Affiliated Hospital of Xi’an Jiaotong University, Xi’an, Shaanxi, China; 6 Department of Cancer Genetics, Roswell Park Cancer Institute, Buffalo, NY, USA; 7 Department of Medical Oncology, The First Affiliated Hospital of Xi’an Jiaotong University, Xi’an, Shaanxi, China; 8 Department of Biology and Biochemistry, University of Houston, Houston, TX, USA

## Abstract

The cancer stem cell (CSC) theory depicts a special population within the cancer mass that self-renew and sustain the cancer, even if the other cells were eliminated by therapies. How CSCs acquire these unique traits is still unclear. Crumbs homolog 3 (CRB3), a member of the CRB polarity complex, has been reported to act as a tumor suppressor. Here, we detected significantly lower or negative CRB3 expression in human breast cancer tissues. Knockdown of CRB3 generated non-tumorigenic, immortalized breast epithelial cell line MCF 10A with CSC properties. Simultaneously, we found that CRB3 downregulation induced the epithelial–mesenchymal transition and activated TAZ (transcriptional co-activator with PDZ-binding motif) and β-catenin. Significantly, the activation of TAZ and β-catenin sufficed in conferring MCF 10A cells with CSC properties. This study demonstrates that cell polarity proteins may serve as a switch of the differentiated vs multipotent states in breast cancers.

## Introduction

The dysregulation of cell polarity proteins plays an important role in cancer development. The establishment and maintenance of epithelial polarity primarily depends on three cell polarity complexes, namely the Crumbs (CRB) complex, the partitioning defective (PAR) complex and the Scribble (SCRIB) complex.^1^

Of all the human Crumbs isoforms, only CRB3, one of the three human Crumbs isoforms (CRB1–3) that is localized to the apical membrane, is widely expressed in the epithelium.^2, 3^ Whiteman *et al.* found that CRB3 was essential for proper epithelial development and viability. CRB3 knockout mice died shortly after birth and displayed epithelial morphogenesis defects such as cystic kidneys, proteinaceous debris throughout the lungs, villus fusion and apical membrane blebs in the intestines.^4^ Specifically, CRB3 repression disrupted tight junction (TJ) formation, while CRB3 increased the expression of the TJ proteins occludin and ZO-1.^5, 6, 7, 8, 9, 10, 11^

The role of CRB3 in epithelial polarity may suggest its role as a tumor suppressor.^7^ Our previous study has found that CRB3 was weakly expressed in clear cell renal cell carcinoma tissues and was a prognostic indicator of clear cell renal cell carcinoma.^12^ CRB3 downregulation in the mouse kidney epithelium disrupted epithelial polarity, increased cell migration and metastasis and resulted in tumorigenicity.^7^ CRB3 knockdown induced the Eph4 mouse mammary epithelial cells to undergo transforming growth factor-β-mediated epithelial–mesenchymal transition (EMT),^13^ suggesting that CRB3 may possess tumor suppressive potential in human mammary epithelial cells.

One hypothesis posits that cancer initiation and progression are triggered by cancer stem cells (CSCs).^14^ CSCs are defined as a small subpopulation of cancer cells specifically endowed with the ability of self-renewal, a process that drives tumorigenesis and differentiation.^15, 16^ Malignant progression is generally accompanied by an increased proportion of CSCs^17^ and initiation of EMT of neoplastic cells.^16, 18^ In the EMT process, epithelium loses various epithelial characteristics, such as junctions, and begins to exhibit mesenchymal characteristics, including migration and invasion. EMT is a complex transdifferentiation program that endows human mammary epithelial cells and tumor cells with stemness properties.^19, 20^

Recent studies have shed new light on the molecular mechanisms of CSCs by discovering cell polarity proteins in inducing EMT.^21^ In this study, we examined how CRB3 expression affects the propensity for MCF 10A cells to exhibit a CSC phenotype.

## Results

### The expression pattern of CRB3 in breast cancer cell lines and clinical breast cancer tissues

**Figure 1:**
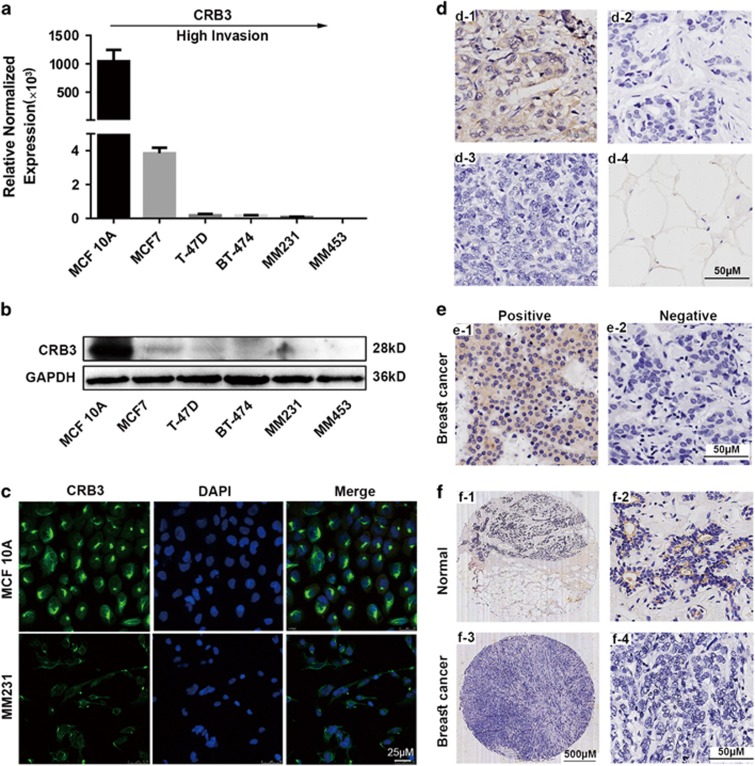
CRB3 expression pattern in breast cancer cell lines and clinical breast cancer tissues. (**a**) CRB3 mRNA expression in breast cell lines evaluated by real-time PCR, relative to MM453 expression. (**b**) Western blot of CRB3 in these cell lines. (**c**) CRB3 localization as shown by IF. (**d**) CRB3 was expressed in the (d-1) breast cancer tissue but was not expressed in the (d-2) breast cancer tissue incubated with PBS in place of primary antibody, in the (d-3) breast cancer tissue incubated with CRB3 recombinant protein and primary antibody rabbit polyclonal anti-CRB3 antibody or in the (d-4) adipose tissue. d-2, d-3 and d-4 were used as negative controls. (**e**) Examples of positive and negative CRB3 expressions in breast tissue microarrays (TMAs). The score of the positive example was 3 (intensity of the staining) *2 (extent of the staining). The score of the negative example was 0 (intensity of the staining) *0 (extent of the staining). (**f**) Examples of high (top) and low (bottom) CRB3 expressions in breast TMAs. Normal, adjacent breast tissues.

To investigate CRB3 expression in human mammary epithelial cells, we examined *CRB3* mRNA and protein levels in a non-tumorigenic cell line (MCF 10A) and five breast cancer cell lines (MCF7, T-47D, BT-474, MDA-MB-231(MM231) and MDA-MB-453(MM453)). Real-time PCR and western blot showed that MCF 10A had the highest *CRB3* mRNA and protein expression among all the cell lines (Figures 1a and b). *CRB3* mRNA expression in MCF 10A cells was remarkably 10^5^-fold that of the tumor cells. Among the breast cancer cell lines, MCF7 showed the higher CRB3 mRNA and protein expression levels, followed by T-47D, BT-474, MM231 and MM453, in descending order. These expression patterns are consistent with the reported role of CRB3 as a tumor suppressor. We also studied the localization of CRB3 in human mammary epithelial cells. CRB3 was localized to the apical membrane in confluent MCF 10A cells, but was excluded from this area and was predominantly cytoplasmic in confluent MM231 cells (Figure 1c).

We next determined CRB3 expression levels in normal breast and breast cancer tissues by immunohistochemistry. Figure 1d showed positive and negative controls of CRB3 antibody. Figure 1e showed examples of positive and negative CRB3 expressions in breast tissue microarrays. Adjacent breast tissues showed uniform and strong CRB3 staining in the apical cytomembrane of the epithelial cells compared with the breast cancer tissues (Figure 1f). CRB3 was localized to the apical membrane in normal breast tissues, but predominantly localized to the cytoplasm in breast cancer tissues. These findings are in the same ballpark as the results of previous reports, that is, CRB3 is localized to the lumenal side of the epithelia in kidney and lung epithelia.^22, 23, 24^ Furthermore, statistically significant CRB3 downregulation was detected in breast cancer tissues (9/41, 22.0%) in comparison with adjacent breast tissues (30/41, 73.2%) (Table 1). Supplementary Table S1 shows the relationship between CRB3 expression and various clinicopathological parameters of breast cancer patients. A significant negative association was found between CRB3 expression and tumor size in breast cancer tissues (Supplementary Table S1). These data indicate that CRB3 may be involved in tumorigenesis and cell growth of human breast cancer.

### CRB3 downregulation promotes CSC characteristics in MCF 10A cells

**Figure 2:**
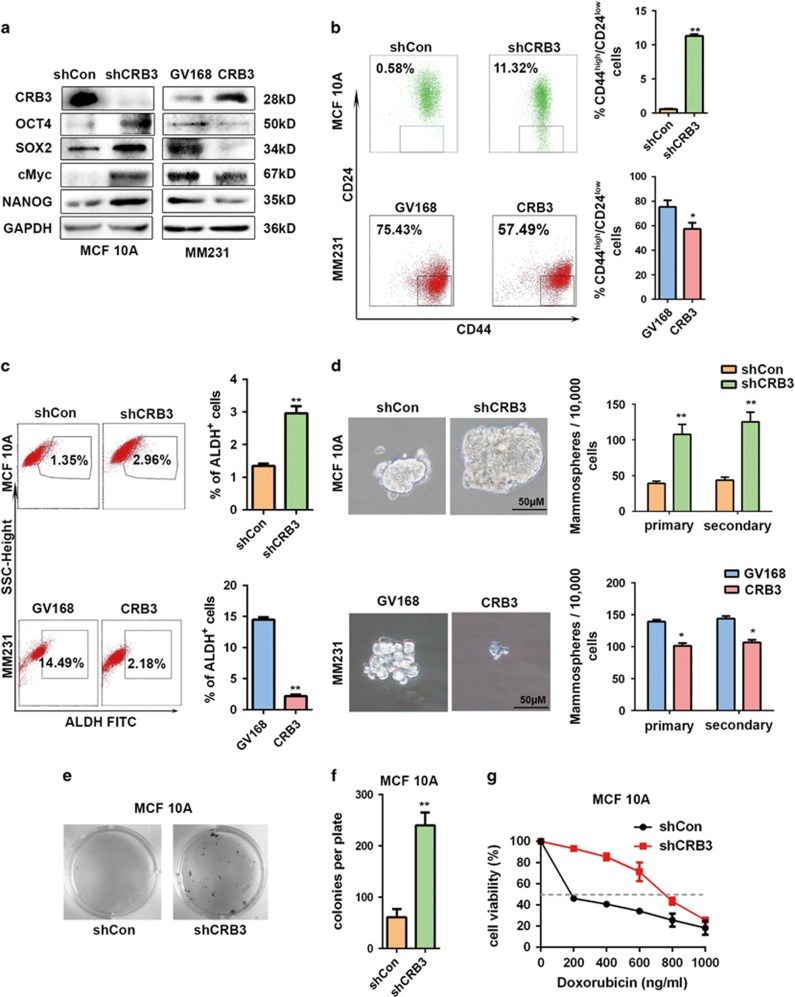
Downregulation of CRB3 enhances breast CSC properties. (**a**) Western blot of CRB3 and CSC markers. (**b**) Fluorescence-activated cell sorting (FACS) profiles and quantification of the CD44^high^/CD24^low^ population. (**c**) FACS profiles and quantification of the stem cell marker ALDH. (**d**) Representative images and quantification of the formed mammospheres; the bar represents 100 μm. (**e**) Representative images of colony formation in soft agar that are quantified in (**f**). (**g**) CRB3 downregulation confers partial drug resistance. All data are presented as mean±s.e.m. and statistical significance was calculated using a two-tailed *t*-test.

To gain initial knowledge if CRB3 is important for ‘stemness’, we altered CRB3 expression in MCF 10A cells and examined the protein expression levels of the CSC-TFs (transcription factors) SOX2, cMyc, OCT4 and NANOG^25^ by western blot (Figure 2a). We found that CRB3 knockdown upregulated CSC-TFs in MCF 10A breast epithelial cells, whereas CRB3 overexpression supressed CSC-TFs in MM231 breast cancer cells. These data show an inversed association between CRB3 and CSC-TFs.

CD44^high^/CD24^low^ and ALDH immunophenotypical cells represent a more differentiated tumor progeny of limited stemness potential.^21, 26^ We hence evaluated these markers upon manipulation of CRB3 expression. The fluorescence-activated cell sorting analysis revealed that the CD44^high^/CD24^low^ and ALDH^+^ subpopulations were significantly increased upon CRB3 knockdown and were decreased upon CRB3 overexpression (Figures 2b and c and Supplementary Figures S1A and B). Strikingly, in cells with downregulated CRB3, a nearly 20-fold increase in the CD44^high^/CD24^low^ population was observed (*P*<0.001) (Figure 2b). The EGF-supplemented serum-free mammosphere formation is a standard assay of CSC self-renewal.^27^ Here, CRB3 knockdown resulted in an increase in the size and number of mammospheres in MCF 10A (Figure 2d) and MCF 12A cells (Supplementary Figures S1C and D). Consistently, MCF 10A cells transformed with shCRB3 displayed strikingly increased colony sizes in soft agar (Figures 2e and f).

In addition to self-renewal, another characteristic of CSCs is their capacity to resist chemotherapy.^21^ We noticed that CRB3-knockdown MCF 10A cells were more resistant than control cells to chemotherapeutic drug doxorubicin (Figure 2g). Taken together, these results indicate that CRB3 downregulation promoted CSC features in MCF 10A cells.

### CRB3 downregulation disrupts MCF 10A cell epithelial polarity

**Figure 3:**
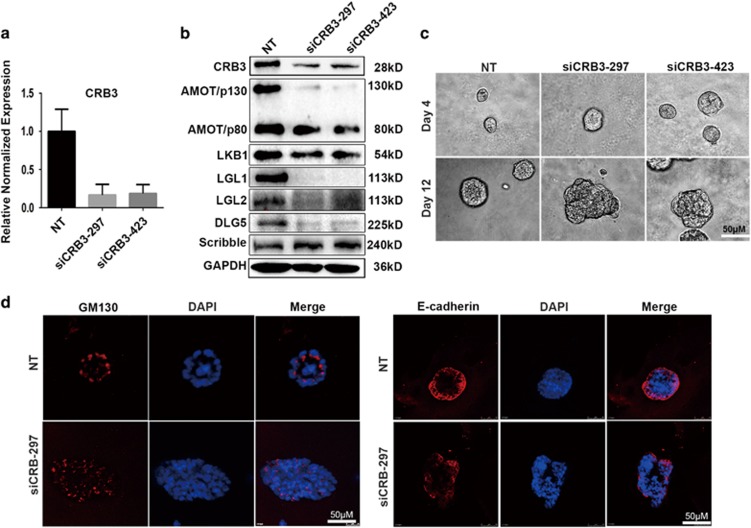
CRB3 downregulation results in morphological alteration and dysregulation of apical–basal polarity. (**a**) MCF 10A cells were transfected with NT, siCRB3-297 or siCRB3-423. The CRB3 levels were detected by real-time PCR. (**b**) Western blot detecting the expression of other polarity proteins after CRB3 downregulation. (**c**) Morphogenesis of MCF 10A cells plated on matrigel. (**d**) NT- or siCRB3-297-transfected MCF 10A cells stained with GM130 (Golgi marker) and E-cadherin on day 12.

To gain insight into the role of CRB3 in epithelial cell polarity maintenance, we performed a three-dimensional (3D) morphogenesis experiment using control- or CRB3-knockdown MCF 10A cells (Figure 3a). The expression of other polarity proteins was determined to explore the possible mechanism underlying the role of CRB3 in epithelial organization. We found that CRB3 downregulation resulted in reduced levels of other apical polarity proteins including the 130 kDa isoforms of AMOT (AMOT/p130), LKB1, LGL1, LGL2 and DLG5 (Figure 3b).

To gain insight into the role of CRB3 in maintenance of epithelial cell polarity, we performed the 3D morphogenesis experiment and the results are shown in Figures 3c and d. After being cultured for 12 days, normal MCF 10A acini possessed a spherical architecture of centrally located apoptotic cells,^28^ while 50% of the CRB3-knockdown cells formed acinar structures larger in size or complex multiacinar structures without apoptosis of glandular lumen cells (Figure 3c). These heterogeneous acinar structures were easily distinguishable from those formed by control cells, indicating that CRB3 downregulation disrupted the epithelial polarity of MCF 10A cells cultured in 3D. Polarity was also assessed using the localization of Golgi marker GM130^29, 30, 31^ and epithelial marker E-cadherin.^32, 33^ The orientation of the Golgi apparatus was monitored by staining GM130, a cytosolic coiled-coil protein anchored to Golgi membranes. During the 3D morphogenesis assay, the Golgi were always oriented toward the lumen of the control acini, whereas the acini formed by CRB3-knockdown MCF 10A cells displayed a modest but reproducible disruption of Golgi orientation (Figure 3d). Meanwhile, epithelial marker E-cadherin staining showed mislocalization and discontinuous distribution of E-cadherin in MCF 10A cells (Figure 3d). These results suggest that CRB3 downregulation disrupted the epithelial organization of MCF 10A cells.

### CRB3 downregulation induces EMT and promotes migration and invasion of human mammary epithelial cells

**Figure 4:**
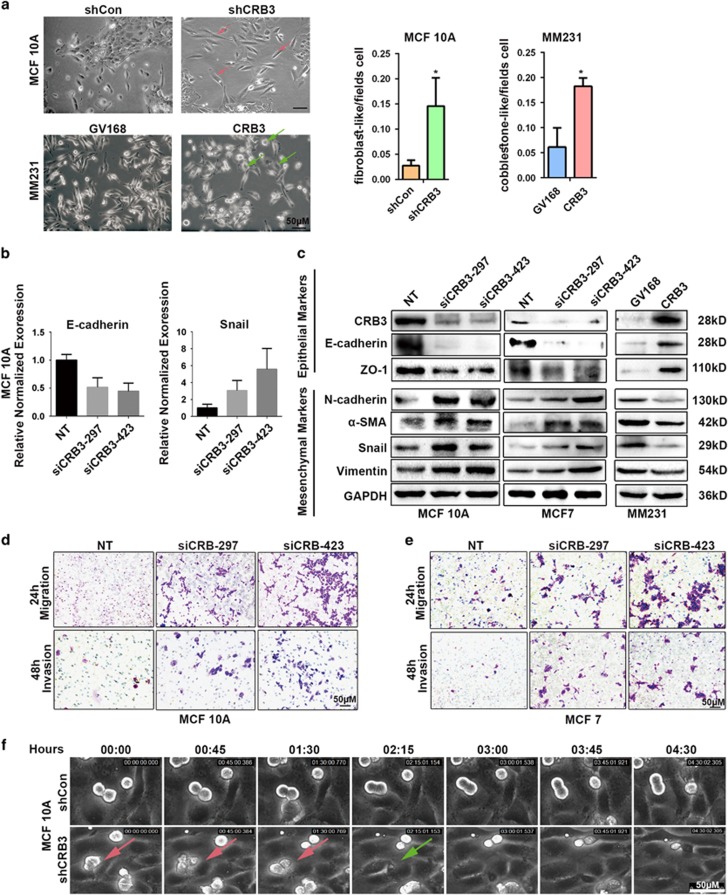
CRB3 downregulation induces EMT program and promotes migration and invasion in human breast cells. (**a**) Morphogenesis and quantification of MCF 10A cells transduced with shCRB3 and MM231 cells infected with CRB3, shown by an inverted phase-contrast microscope (red arrows or green arrows to indicate which cells acquire fibroblast- or cobblestone-like). (**b**) E-cadherin and Snail expressions by real-time PCR. (**c**) CRB3 and EMT marker expression levels assessed by western blot. (**d**) Cell migration and invasion assays showing migration and invasion of MCF 10A and (**e**) MCF7 cells transfected with NT and siCRB3. (**f**) Suppression of transendothelial migration. All data are presented as mean±s.e.m. and statistical significance was calculated using a two-tailed *t*-test.

Maintaining epithelial polarity is crucial for preventing EMT, which is a complex process closely related to CSC properties and cancer invasion/metastasis.^19, 20^ Thus, we next determined if CRB3-knockdown had an effect on EMT. Compared with control cells, CRB3-knockdown MCF 10A cells demonstrated a dramatic change in morphology, with transformation of the cobblestone-like epithelial morphology to an elongated fibroblast-like morphology accompanied by pronounced cellular scattering (Figure 4a). Consistently, we found that CRB3-knockdown resulted in a significant upregulation of Snail and downregulation of E-cadherin in MCF 10A cells by real-time PCR (Figure 4b). Xaralabos Varelas *et al.*^13^ also found that Snail were increased after CRB3 downregulation. In contrast, CRB3 overexpression caused some fibroblast-like MM231 cells to become cobblestone-like (Figure 4a). Agreeing to morphological changes, CRB3-overexpressing MM231 cells showed increased expressions of epithelial markers and decreased expression of mesenchymal markers (Figure 4c). These data support that loss of CRB3 is a crucial step in initiating EMT in human mammary epithelial cells.

Next, we performed the transwell assay to test if CRB3-knockdown affects cell migration/invasion (Figures 4d and e). In this transwell assay, a layer of human umbilical vein endothelial cells (HUVEC) serve as the barrier for MCF 10A cells to penetrate. In continuous live image monitoring, CRB3-knockdown MCF 10A cells started to migrate across the layer of HUVEC at ~0 h 45 min (as indicated by the pink arrowhead in the lower panel), and reached the other side at ~2 h 15 min (as indicated by the green arrowhead in the lower panel). In contrast, control cells did not migrate through the HUVEC layer at ~4 h 30 min, when the live monitoring was ended. These results indicate that loss of CRB3 may increase the migration and invasion ability of breast cells (Figure 4f).

### CRB3 downregulation induces TAZ and β-catenin activation in MCF 10A cells

**Figure 5:**
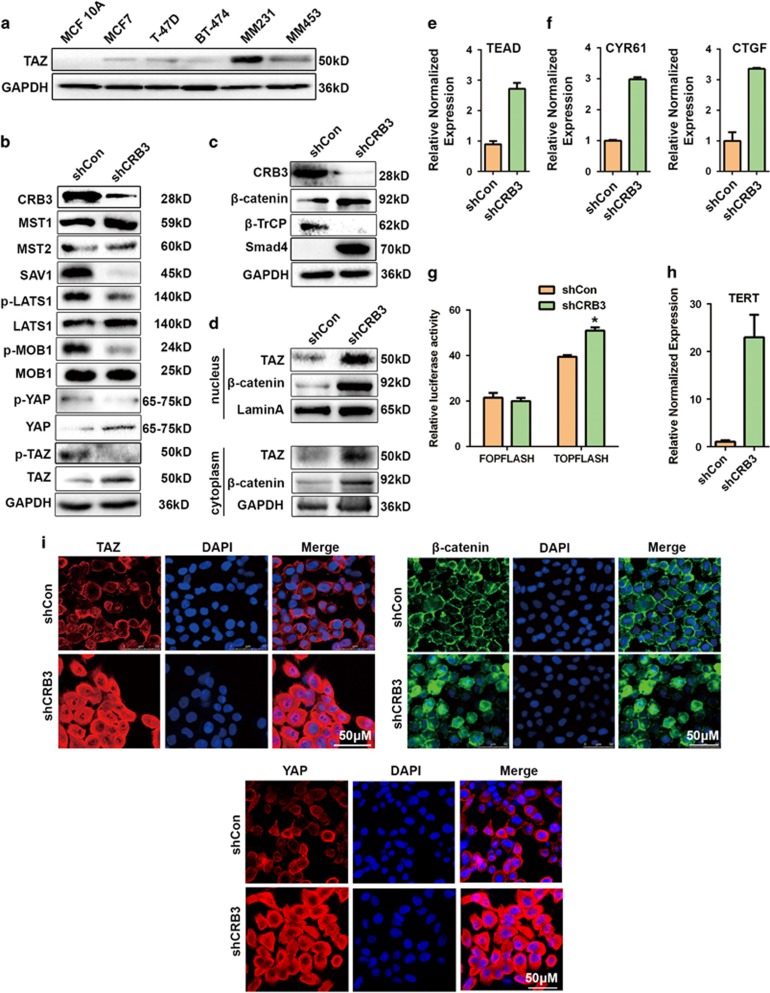
CRB3 downregulation induces activation of TAZ and β-catenin. (**a**) Western blot of CRB3 in human mammary epithelial cells. (**b**) Western blot of the Hippo pathway components. (**c**) Western blot of β-catenin, β-TrCP and βTrcp substrates Smad4 after CRB3 knockdown. (**d**) Cytoplasmic and nuclear expression of TAZ and β-catenin. (**e**) MCF 10A cells were transfected with the TEAD-luciferase reporter and TEAD promoter activity was detected by luciferase assay. (**f**) Real-time PCR showed that CRB3 knockdown increased expressions of TAZ target gene CYR61 and CTGF. (**g**) Luciferase assay of TOPFLASH or control FOPFLASH as a measure of β-catenin/TCF activity. (**h**) Real-time PCR of TERT. (**i**) Localization of TAZ, β-catenin and YAP as shown by IF. All data are presented as mean±s.e.m. and statistical significance was calculated using a two-tailed *t*-test.

Works in *Drosophila* have linked the Crumbs proteins to the Hippo pathway, a key regulator of organ size. The Hippo transducer TAZ was also found to confer CSC traits on breast cancer cells.^21^ We found that luminal breast cancer cells possessed higher levels of TAZ compared with MCF 10A cells, while TAZ expression was higher in basal-like breast cancer cells than in luminal breast cancer cells (Figure 5a). Integrating with our findings thus far, we hypothesize that the CSC properties in CRB3-knockdown cells is mediated by TAZ activation. Upon CRB3 knockdown, we detected a decrease in SAV1 protein expression and the phosphorylation of LATS1, MOB1 and YAP, together with a significant decrease in p-TAZ and increase in TAZ expression (Figure 5b). TAZ plays a cytoplasmic inhibitory role in Wnt/β-catenin signaling,^34^ which, in turn, plays a prominent role in stem cell maintenance.^35, 36, 37, 38^ We found that β-catenin was increased after CRB3 knockdown (Figure 5c). Phosphorylated TAZ is associated with the β-TrCP E3 ubiquitin-ligase complex,^39^ which otherwise triggers degradation of phosphorylated β-catenin.^40^ Crucially, CRB3 knockdown resulted in a decrease in β-TrCP and β-TrCP substrates Smad4 expressions (Figure 5c). Increased TAZ and β-catenin expression was accompanied by altered subcellular distribution. β-Catenin, TAZ and YAP were predominantly localized in the nucleus in CRB3-knockdown MCF 10A cells (Figures 5d and i). In line with TAZ and β-catenin expression patterns, luciferase assays showed increased TEAD transcriptional activity (upstream of TAZ) (Figure 5e) and increased CTGF and CYR61 (downstream of TAZ) mRNA expressions (Figure 5f), indicating that the Hippo pathway is in the activation mode. Likewise, the Wnt reporter TOPFLASH was stimulated (Figure 5g) and Wnt/β-catenin signaling pathway downstream target gene TERT was increased (Figure 5h).^41^ Thus, our data are consistent with a notion that CRB3 downregulation promoted a cascade of TAZ and Wnt/β-catenin activation.

### CRB3 downregulation confers CSC traits in breast cancer cells through the TAZ/β-catenin cascade

**Figure 6:**
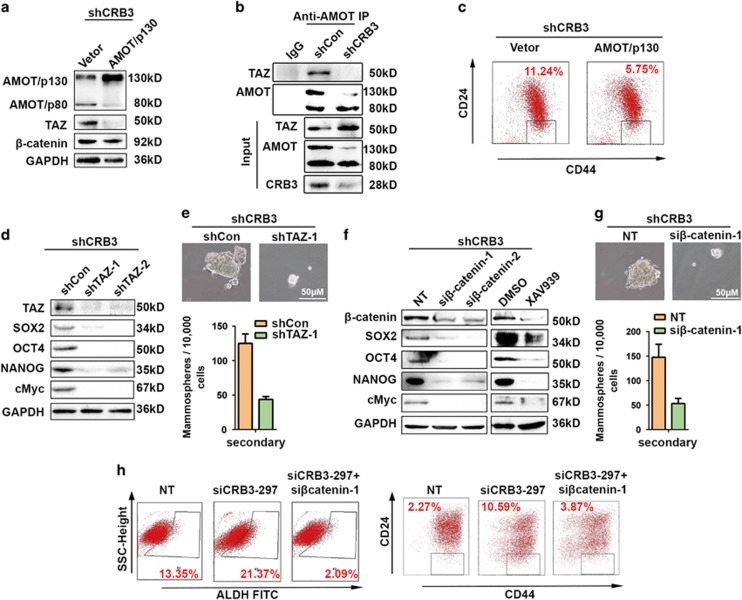
Downregulation of CRB3 confers CSC traits on breast cancer cells through the TAZ/β-catenin cascade. (**a**) Western blot detecting the expressions of AMOT, TAZ and β-catenin. (**b**) CoIP/western blot analysis showing endogenous AMOT bound to TAZ. (**c**) Fluorescence-activated cell sorting of CD44^high^/CD24^low^ population. (**d**) Western blot of TAZ and stem cell markers. (**e**, **g**) Representative images and quantification of formed mammospheres. (**f**) Western blot of β-catenin and stem cell markers. XAV939 was used to inhibit β-catenin. (**h**) Flow cytometry analysis of the stem cell marker ALDH and the CD44^high^/CD24^low^ population. All data are presented as mean±s.e.m. and statistical significance was calculated using a two-tailed *t*-test.

TAZ was demonstrated as an inducer of breast CSC traits.^21, 42^ Here, we set to determine if CRB3 attenuation mediates the effect of TAZ. AMOT/p130 was found to specifically interact with TAZ and sequester TAZ in the cytoplasm.^43, 44, 45^ Therefore, we tested whether AMOT/p130 abolishes CSC traits in MCF 10A cells. As shown in Figure 6a, AMOT/p130 overexpression in CRB3-knockdown MCF 10A cells resulted in a robust downregulation of TAZ protein levels but did not affect the β-catenin level. The AMOT/p130 and TAZ complex was damaged after CRB3 downregulation due to downregulation of AMOT/p130 (Figure 6b). Notably, the CD44^high^/CD24^low^ subpopulation was reduced after the AMOT/p130 level was ectopically restored (Figure 6c), suggesting that manipulation of the Hippo pathway by AMOT/p130 reduces the CSC population in CRB3-knockdown MCF 10A cells.

To further establish whether CRB3 acted through TAZ and β-catenin to confer CSC traits, we knocked down TAZ and β-catenin by siRNAs, or used a β-catenin inhibitor XAV939 (Selleck) to inhibit the Wnt pathway. Compared with controls, TAZ knockdown, β-catenin knockdown, or XAV939 each markedly suppressed the expression levels of the CSC markers SOX2, OCT4, NANOG and cMyc (Figures 6d and f). Furthermore, the number and size of the mammospheres were significantly decreased (Figures 6e and g). We also confirmed that β-catenin downregulation reduced the ALDH^+^ and CD44^high^/CD24^low^ subpopulations in CRB3-knockdown MCF 10A cells (Figure 6h).

### CRB3 upregulation reduces tumorigenic potential of breast cancer cells *in vivo*

**Figure 7:**
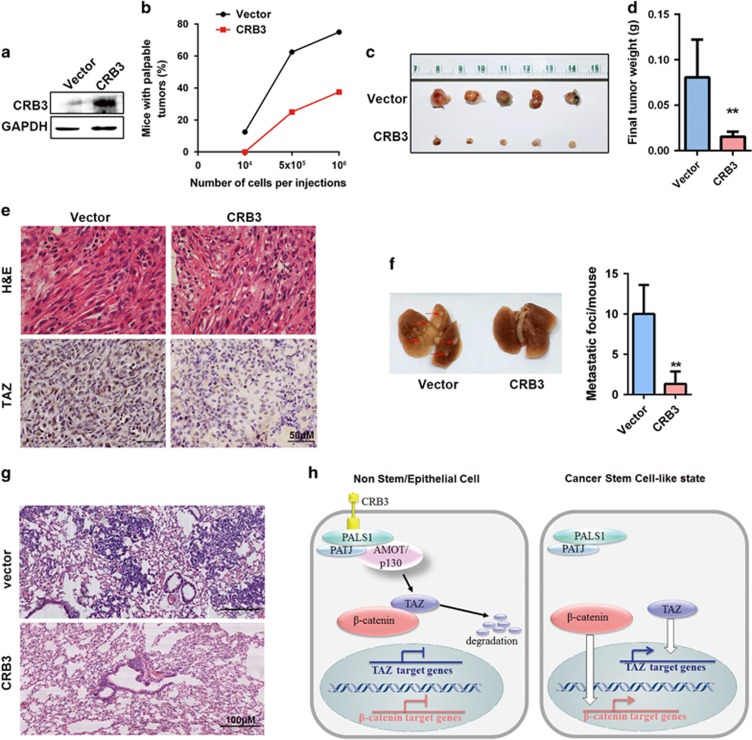
CRB3 upregulation reduced tumorigenic potential of MM231 cells *in vivo*. (**a**) The CRB3 levels were detected by western blot. (**b**) Tumor-seeding ability of MM231-Vector and mm231-CRB3 cells. (**c**) Representative tumor pictures are shown. (**d**) Tumor weight was evaluated on the 25 day. (**e**) Immunohistochemistry staining of tumor specimens. (**f**) Visualized lung metastasis models after orthotopic injection (arrows: metastatic foci) and the number of metastatic foci was counted as nodules. (**g**) Hematoxylin and eosin staining of lung metastasis specimen. (**h**) Schematic of the proposed epistatic relationships between CRB3, TAZ, β-catenin and CSC traits. All data are presented as mean±s.e.m. and statistical significance was calculated using a two-tailed *t*-test.

To verify CRB3 effects on CSCs *in vivo*, we injected Vector-infected MM231(left) or CRB3-overexpressing MM231(right) cells into the fat pad of severe combined immunodeficiency mice (Figure 7a). Twenty-four mice were divided randomly into three groups. As shown in Figure 7b, palpable tumor masses developed in mice injected with >5 × 10^5^ both cells, but only control cells formed tumors when as few as 10^4^ cells were injected. Remarkably, the reduced tumor-initiation capacity of MM231-CRB3 cells was accompanied by reduced size, weight and histological grade of the tumors (Figures 7c–e). Control MM231 cells induced the formation of invasive carcinomas showing high nuclear pleomorphism and prominent nucleoli. Conversely, CRB3-overexpressing cells formed carcinomas displaying less nuclear atypia (Figure 7e). To verify CRB3 effects on invasive potential, 16 four-week-old female mice were divided randomly into two groups and 10^6^ MM231 cells containing Vector or CRB3 were injected into the right fat pad of the mice. We found that CRB3-overexpressing cells formed less pulmonary metastasis (Figures 7f and g). Thus, CRB3 upregulation reduced the tumorigenic potential of MM231 cells and the acquisition of a less malignant and more differentiated phenotype.

In addition, TAZ expression was significantly decreased in CRB3-overexpressing cells *in vivo* (Figure 7e), which is consistent with the results of the *in vitro* experiments. These data suggest a scenario in which CRB3 knockdown led to EMT, which activated TAZ, relieved β-catenin from TAZ inhibition and ultimately promoted CSC-related traits (Figure 7h).

## Discussion

In this study, we found that CRB3 was strongly expressed in normal breast epithelial tissues, but weakly expressed in breast cancer tissues. CRB3 expression is negatively associated with tumor malignancy. These data suggest that CRB3 may be a cancer suppressor, which is consistent with Karp and his colleagues’ previous findings that CRB3 expression correlates inversely with migration and invasion of the mouse kidney epithelial cells and that reduced expression of CRB3 can promote carcinogenesis of murine kidney epithelia.^7^ In human mammary epithelial cells, CRB3 downregulation was associated with stem cell molecular signatures, increased EMT and invasion potential, which are considered as hallmarks of CSC activity. Taken together, our study suggests that CRB3 downregulation is associated with human breast CSCs. At the functional level, we showed that increased CRB3 levels inhibited CSC-related traits *in vivo* and *in vitro*.

As a hyperactive downstream effector protein of the Hippo pathway, TAZ may promote tumorigenic potential by enhancing stem cell-like properties in breast cancer.^21^ CRB3 downregulation has been shown to be associated with TAZ localization in the nucleus.^7^ We showed that the expression of TAZ and other downstream factors were increased in CRB3-downregulated cells. However, further research is required to elucidate the precise mechanism by which CRB3 inhibits the function of Hippo signaling. Our results demonstrated that CRB3 downregulation disrupted the epithelial organization of MCF 10A cells in the 3D culture. CRB3 downregulation decreased the expression of the polarity protein AMOT/p130, which has been found to bind to PATJ and PALS1, members of the CRB complex.^46^ AMOT/p130 was able to form a complex with TAZ, and we found that CRB3 downregulation reduced AMOT/p130 but increased nuclear TAZ levels. When the Hippo pathway is active, YAP and TAZ are phosphorylated. Our data showed that p-TAZ was decreased, whereas nuclear TAZ was increased. Furthermore, TAZ has been shown to play a cytoplasmic inhibitory role in the Wnt/β-catenin signaling pathway.^34^ Nuclear YAP/TAZ may trigger ‘Wnt’ signaling, perhaps even in the absence of Wnt ligands.^47^ Therefore, the nuclear β-catenin was upregulated by increased nuclear TAZ. We speculated that CRB3 regulated TAZ through AMOT/p130, and our results suggest that TAZ/β-catenin was the key downstream targets of CRB3, whose activities sustained the self-renewal and tumor-initiation capacities of breast CSCs.

This study is the first to investigate the relationship between CRB3 expression and the clinicopathological features of breast cancer patients. Only 41 breast cancer samples were used in this study. The relationship between CRB3 and clinical or histological stage was not statistically significant. A further study using a larger sample size is needed to verify the relationship between CRB3 and clinical or histological stage. Furthermore, we demonstrated that removal of a single protein of the polarity complex is sufficient in inducing CSC traits in breast epithelial cells. Liu *et al.* discovered through fluorescence-activated cell sorting that BCSCs exist in distinct mesenchymal-like (EMT) and epithelial-like (MET) states. Mesenchymal-like BCSCs characterized as CD44^+^/CD24^−^ are primarily quiescent and are capable of invasion, whereas epithelial-like BCSCs expressed ALDH were proliferative.^48^ Our results showed that CRB3 depletion increased both CD44^+^/CD24^−^ and ALDH^+^ cell population and promoted migration and invasion. According to the Liu’s finding, the CD44^+^/CD24^−^ subset may play a leading role in invasion in CRB3 depletion cells. Our results support that CRB3 regulates multiple cellular processes such as epithelial polarity, cell migration and invasion by regulating Hippo signaling. Previously, it was demonstrated that direct perturbation of CRB3 can lead to phenotypic changes that facilitate tumor progression,^13, 23, 49^ our study agree with previous findings, but support that CRB3 most likely affected migration and invasion through CSC regulation. We have established a CRB3–TAZ–β-catenin cascade that confers CSC traits, but the remaining details, especially the molecular connection between CRB3 and TAZ, require further clarification. In the future, understanding of how CRB3 is deregulated during the initiation and progression of breast cancer may lead to new diagnostic and therapeutic opportunities for breast cancer.

## Materials and methods

### Immunohistochemistry and scoring

A total of 41 pairs of breast cancer tissues and their adjacent breast tissues were obtained from the First Affiliated Hospital of Xi’an Jiaotong University and the Shanghai Outdo Biotech Co., Ltd. This study was conducted according to Ethical Committee on Human Research of the First Affiliated Hospital of Xi’an Jiaotong University and written informed consent had been obtained from all patients. The antibody against CRB3 (013835) was obtained from Sigma-Aldrich (St Louis, MO, USA). Each immunohistochemistry image was read and scored by a pathologist twice and at three different microscopic fields each time. The pathologist was blinded to the group allocation.

The intensity of the immunohistochemistry staining was scored as 0 (negative), 1 (weakly positive), 2 (moderately positive) or 3 (strongly positive). The extent of the staining was defined as the percentage of stained cells per field and scored as 0 (negative), 1 (1–50%) or 2 (51–100%). The staining score for each field was calculated as the product of the intensity and extent of the staining. The expression level was considered negative if the staining score was 0–2 and positive if the staining score was 3–6.

### Cell culture, transfection and infection

MCF 10A and MCF 12A non-tumorigenic human mammary epithelial cells were cultured as previously described.^33^ Human breast cancer cells MCF7, T-47D, MM231 and MM453 were cultured in Dulbecco's modified Eagle's medium (GE, Pittsburgh, PA, USA) supplemented with 10% fetal bovine serum (GE), and the BT-474 cell line was cultured in the RPMI-1640 medium (GE) supplemented with 10% fetal bovine serum. All cells were maintained at 37 °C in a humidified atmosphere containing 5% CO_2_. MCF 10A and MCF 12A cell lines were given by Jianmin Zhang. Other cell lines were obtained from Shanghai Institute of Cell Biology (Shanghai, China) in the Chinese Academy of Sciences. All cell lines have never been passaged longer than 3 months and the cell lines were characterized by Genetic Testing Biotechnology Corporation (Suzhou, China) using short tandem repeat markers.

CRB3 Lentivirus, siRNA and shRNA were purchased from GenePharma Company (Shanghai, China). The CRB3 siRNA sequences (5′–3′) were as follows: CRB3-297 sense, AUG AGA AUA GCA CUG UUU UTT; CRB3-423 sense, UGG CAC UGU UGG UGC GGA ATT. The β-catenin siRNA sequences (5′–3′) were as follows: β-catenin-1 sense, AUG AGA AUA GCA CUG UUU UTT; β-catenin-2 sense, UGG CAC UGU UGG UGC GGA ATT. The shRNA sequences were (5′–3′) as follows: shCRB3, GGG CAA ATA CAG ACC ACT TCT; Control-shRNA (shCon), TTC TCC GAA CGT GTC ACG T. shTAZ were a gift from Jianmin Zhang.

### Real-time PCR

The primers were designed by TaKaRa (Dalian, China). Measurements were performed in triplicate and standardized to glyceraldehyde 3-phosphate dehydrogenase levels. Primer pairs used in real-time PCR are listed in Table 2.

### Western blot and immunoprecipitation

CRB3 antibody (292449), SOX2 antibody (17320), cMyc antibody (764) and p-TAZ antibody (17610) were purchased from Santa Cruz (Dallas, TX, USA); NANOG antibody (55241) was purchased from Sangon Biotech (Shanghai, China); OCT4 antibody (WL1005a) was purchased from Wanleibio (Shenyang, China); Scribble antibody (4475), LKB1 antibody (3050), β-catenin antibody (9562 s), Snail antibody (3879); β-TrCP antibody (4394); Smad4 antibody (12747) and the Hippo signaling antibody sampler kit (8579) were purchased from Cell Signaling (Beverly, MA, USA); Vimentin antibody (10366), LaminA antibody (10298) and GAPDH antibody (HRP-60004) were obtained from Proteintech (Wuhan, China); E-cadherin antibody (610405) and N-cadherin antibody (610920) were purchased from BD Biosciences (San Jose, CA, USA); α-SMA antibody (A2547) and DLG5 antibody (000555) were purchased from Sigma-Aldrich; LGL1 antibody (H00003996-M01) and LGL2 antibody (H00003993-M06) were purchased from Abnova (Taiwan, China); TAZ antibody (MAB7210) was obtained from R&D Systems (Minneapolis, MN, USA); ZO-1 antibody (339100) was obtained from ThermoFisher Scientific (Carlsbad, CA, USA); The AMOT antibody was produced by Genemed Synthesis, Inc. (South San Francisco, CA, USA).

Immunoprecipitation (IP) experiments were conducted using protein A-sepharose-bound anti-AMOT monoclonal antibody (Genemed Synthesis, Inc.) according to the Dynabeads Protein A Immunoprecipitation Kit manual.

### Immunofluorescence

Cells were fixed in 4% paraformaldehyde, permeabilized with 0.1% Triton X-100 and stained with the following primary antibodies anti-CRB3 (HPA013835; Sigma-Aldrich), anti-TAZ (MAB7210; R&D Systems), anti-β-catenin (9562 s; Cell Signaling), anti-GM130 (610822; BD Biosciences) and anti-E-cadherin (610405) overnight at 4 °C.

### 3D morphogenesis assay

MCF 10A cells were cultured in Growth Factor Reduced BD Matrigel (354230) in a four-well chamber slide (177437; Corning, Corning, NY, USA) as previously described.^33^

### Fluorescence-activated cell sorting analysis

One million cells were stained with anti-CD44-APC conjugate (103008; Biolegend, San Diego, CA, USA) and anti-CD24-PE conjugate (311118; Biolegend).

The ALDEFLUOR kit (01700; Stem Cell Technologies, Vancouver, BC, Canada) was used to detect intracellular ALDH enzyme activity.

### Plasmids

GV168-CRB3 and AMOT/p130 plasmids were purchased from GeneChem Company (Shanghai, China). MM231 cells were transfected with GV168-CRB3 plasmid using TurboFect Transfection Reagent (ThermoFisher Scientific) according to the manufacturer’s instructions.

### Cell migration and invasion

Migration and invasion assays were performed as previously described.^12^

### Time-lapse recording of transendothelial migration

The transendothelial migration assay was performed as previously described.^50^ Briefly, HUVEC cells were cultured to confluency in a 35-mm dish, and 5 × 10^4^ MCF 10A cells with or without shCRB3 lentivirus infection were seeded onto the confluent HUVEC layer. The behavior of MCF 10A cells was monitored for 12 h with a phase-contrast microscope and photographed.

### Soft agar assay

In total, 1 × 10^4^ cells were added to 1.5 ml of growth medium with 0.4% agar and were layered onto 2 ml of 0.5% agar beds in six-well plates. The cells were fed with 1 ml of medium with 0.4% agar every 7 days for 21 days. Next, the colonies were stained with 0.02% iodonitrotetrazolium chloride (Sigma-Aldrich) and photographed. Colonies larger than 50 μm in diameter were counted as positive for growth.

### Mammosphere formation assay

Mammosphere formation assays were performed by plating 1 × 10^4^ cells in serum-free Dulbecco's modified Eagle's medium/F12 media (Gibco, Grand Island, NY, USA) supplemented with EGF (20 ng ml^−1^) and B27 (2%) into ultra-low attachment six-well plates (Corning). Mammospheres were allowed to grow for 6 days. Total mammospheres greater than 80 μm in diameter were counted.

### Luciferase assay

MCF 10A cells grown in 6-cm plates were cotransfected with 50 ng TEAD-luciferase reporter and 20 ng Renila using TurboFect Transfection Reagent, and LEF/TCF-mediated transcriptional activity was measured using the Super8xTOPFLASH reporter plasmid (plasmid #12456; Addgene, Cambridge, MA, USA) with the Super8xFOPFLASH plasmid (plasmid #12457; Addgene) serving as a control.

### Treatments with chemotherapeutic drugs

One day after seeding, Doxorubicin of different concentrations was added and after 48 h, cell viability was measured with the WST-1 (Roche, Basel, Switzerland).

### *In vivo* tumor model

All animal procedures were performed according to the protocol approved by the Institutional Animal Care and Use Committee at Xi’an Jiaotong University. Four-week-old female severe combined immunodeficiency mice (body weight, ~20 g) were purchased from the Laboratory Animal Center of Xi’an Jiaotong University, China. The tumor sizes were measured twice or three times per week using calipers. After a total of 25 days of treatment, the mice were killed, and the primary tumor tissues were immediately removed. For the metastatic tumor model, tumor growth was monitored up to 6 weeks and animals were killed. Primary tumor and lungs were harvested, paraffin embedded, sectioned and stained with hematoxylin and eosin. The investigator was blinded to the group allocation.

### Statistical analysis

Statistical analyses were performed in SPSS software (Version 22, Armonk, NY, USA). The statistical significance between two groups was compared by two-tailed *t*-test or *χ*^2^-test. All data shown are from experiments that were performed at least three times with similar results on each occasion. Investigator was blinded to the group allocation during the all experiments and when assessing the outcomes. All results are expressed as mean±s.e.m. *P-*values <0.05 and <0.001 were considered significant (*) and highly significant (**), respectively.

## Figures and Tables

**Table 1:** CRB3 protein levels in breast cancer and adjacent breast tissues

*Tissue*	*No.*	*Expression*		χ^*2*^	P
		*Negative*	*Positive*		
Normal	41	11	30	21.564	<0.001
Breast cancer	41	32	9		

**Table 2:** Primer pairs used in real-time PCR

*Gene*		*Primer sequences (5′–3′)*
*GAPDH*	F	CTC CTC CAC CTT TGA CGC TG
	R	TCC TCT TGT GCT CTT GCT GG
*CRB3*	F	CTT CTG CAA ATG AGA ATA GCA CTG
	R	GAA GAC CAC GAT GAT AGC AGT GA
*CTGF*	F	AGG TGT GGC TTT AGG AGC AG
	R	TCT TGA TGG CTG GAG AAT GC
*CYR61*	F	TGG AAC TGG TAT CTC CAC ACG
	R	TAC ACT GGC TGT CCA CAA GG
*E-cadherin*	F	GAG TGC CAA CTG GAC CAT TCA GTA
	R	AGT CAC CCA CCT CTA AGG CCA TC
*Snail*	F	CAG ACC CAC TCA GAT GTC AAG AA
	R	GGG CAG GTA TGG AGA GGA AGA
*TERT*	F	ACGGTGTGCACCAACATCTACAA
	R	TCAGAGATGACGCGCAGGA
