# Supplementary material for: CRB3 downregulation confers breast cancer stem cell traits through TAZ/β-catenin
Source: Oncogenesis. 2017 Apr 24;6(4):e322–. doi: 10.1038/oncsis.2017.24 (PMC5520500; doi:10.1038/oncsis.2017.24)
Supplement: Supplementary Material [file oncsis201724x1.docx]

**Fig. S1** CRB3 downregulation enhances CSC properties. (a) Flow cytometry analysis of the CD44^high^/CD24^low^ population. (b) Quantification of the CD44^high^/CD24^low^ population (mean ± SEM of triplicate experiments). (c) Representative images of the formed mammospheres. (d) Quantification of the mammospheres (mean ± SEM of triplicate experiments).

**Table S1.** Relationships between CRB3 expression levels and clinicopathological parameters of breast cancer patients.

| **Variable** | **No.** | **Expression** | | **χ^2^** | ***P*** |
| --- | --- | --- | --- | --- | --- |
|  |  | **Negative** | **Positive** |  |  |
| Age (years) |  |  |  | 1.834 | 0.176 |
| ≥50 | 24 | 21 | 3 |  |  |
| <50 | 17 | 11 | 6 |  |  |
| Histological grade |  |  |  | 0.484 | 0.487 |
| II | 16 | 11 | 5 |  |  |
| III | 24 | 20 | 4 |  |  |
| missing | 1 |  |  |  |  |
| Clinical stage |  |  |  | 1.567 | 0.211 |
| 0~2 | 27 | 19 | 8 |  |  |
| 3 | 14 | 13 | 1 |  |  |
| Tumor size (cm) |  |  |  | 3.876 | 0.049^*^ |
| <2 | 7 | 3 | 4 |  |  |
| ≥2 | 34 | 29 | 5 |  |  |
| Tumor side |  |  |  |  | 1.000^#^ |
| Left | 21 | 16 | 5 |  |  |
| Right | 20 | 16 | 4 |  |  |
| Lymph node involvement |  |  |  |  | 0.130^#^ |
| Negative | 20 | 18 | 2 |  |  |
| Positive | 21 | 14 | 7 |  |  |

^#^ Fisher’s exact test

Movie S1, related to Figure 4. Facilitated transendothelial migration of MCF 10A cells with CRB 3 downregulation.
