# Supplementary figures and images for: CRB3 downregulation confers breast cancer stem cell traits through TAZ/β-catenin
Source: Oncogenesis. 2017 Apr 24;6(4):e322–. doi: 10.1038/oncsis.2017.24 (PMC5520500; doi:10.1038/oncsis.2017.24)

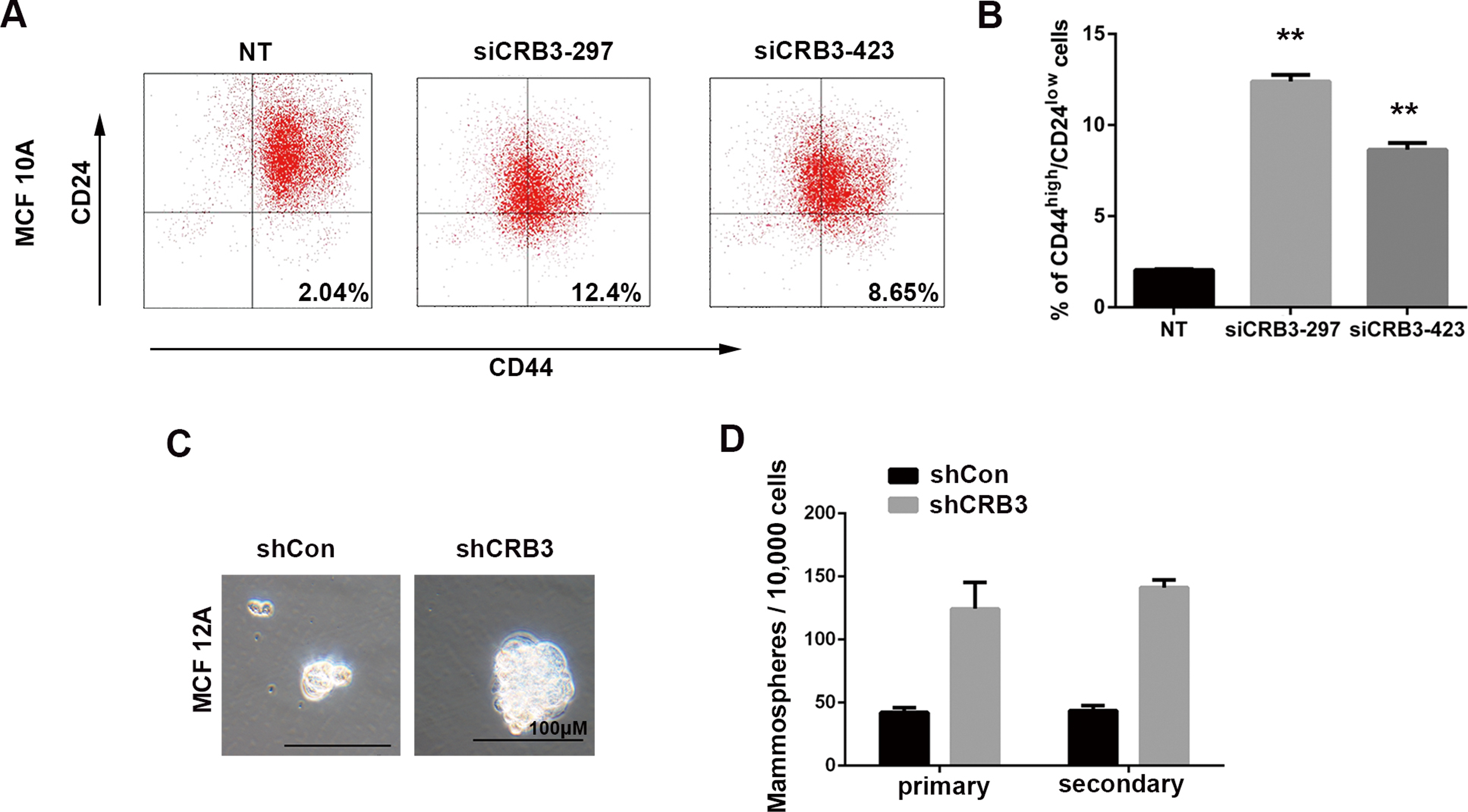

Supplement: Supplementary Figure 1 [file oncsis201724x2.tif]
